# Navigating data governance approvals to use routine health and social care data to evidence the hidden population with severe obesity: a case study from a clinical academic’s perspective

**DOI:** 10.1177/17449871221122040

**Published:** 2022-11-15

**Authors:** Kath Williamson, Amy Nimegeer, Mike Lean

**Affiliations:** NRS Clinician (NHS Lothian), Doctoral Student, Department of Human Nutrition, School of Medicine, Dentistry and Nursing, University of Glasgow, Glasgow, UK; Research Associate (MRC/CSO Social and Public Health Sciences Unit), University of Glasgow, Glasgow, UK; Professor of Human Nutrition, Department of Human Nutrition, School of Medicine, Dentistry and Nursing, University of Glasgow, Glasgow, UK

**Keywords:** approvals, clinical academic, data governance, routinely-collected data, social care

## Abstract

**Background:**

Front-line professionals are uniquely placed to identify evidence gaps and the way routinely-collected data can help address them. This knowledge can enable incisive, clinically-relevant research.

**Aim:**

To document an example of the real-world approvals journey within the current NHS/Higher Education regulatory landscape, from the perspective of an experienced nurse undertaking doctoral study as a clinical academic.

**Methods:**

An instrumental case-study approach is used to explore the approvals process for a mixed-methods study. Relevant context is highlighted to aid understanding, including introduction of the General Data Protection Regulation and the integration of health and social care services.

**Results:**

Formal approvals by nine separate stakeholders from four different organisations took nearly 3 years, including 15 initial or revised applications, assessments or agreements. Obstacles included: conflicting views on what constitutes ‘research’ or ‘service evaluation’; isolated decision-making; fragmented data systems; multiple data controllers and a changing data governance environment. The dual perspectives of being both clinician and academic using routine data are explored.

**Conclusions:**

Practitioners face a complex approvals process to use data they routinely collect, for research or evaluation purposes. Use of data during the COVID-19 pandemic has demonstrated the need for streamlining of data governance processes. Practical recommendations are outlined.

## Introduction

‘Some groups suffer because their experiences are not made visible in the data’ (Steventon, 2020). Front-line care professionals^1^ can identify priority areas for practice development and research (Carrick-Sen et al., 2016) which may be unseen by policy makers, senior managers and academics. Practitioners routinely process data to enable care, providing a familiarity with at least some of what is held in multiple care systems. Increasingly routine data are recognised as potentially rich data sources for answering research questions (Heslop et al., 2020), whilst opening innovative horizons for both care delivery and research (Scobie and Castle-Clarke, 2019). Yet professionals, particularly Nurses, Midwives and Allied Health Professionals (NMAHPs), can face numerous barriers to engaging in research (Marjanovic et al., 2019) whether experimental, observational or service evaluation.

Clinical academic roles, with both clinical and research components, address the twin problems of researchers struggling to engage overburdened clinical staff (Sheard and Peacock, 2019) and poor translation of research findings into real-world care (Carrick-Sen et al., 2016). This case study explores the recent real-world experience of a NMAHP clinical academic planning a service evaluation, including accessing routinely-collected health and social care data, to document a population which is ‘not made visible in the data’. The overall aim being to improve the evidence base to support future service improvements and research (Finch, 2009).

### Care for housebound adults with severe obesity

The evaluation originated from the first author’s role as a senior community nurse, caring for escalating numbers of housebound adults with severe obesity (body mass index (BMI) ⩾40 kg/m^2^). This population experiences poor clinical outcomes from multiple disabling physical and mental health conditions. Skin breakdown is common, aggravated by type 2 diabetes and lymphoedema, but minimal evidence exists to guide clinical practice (Williamson et al., 2020). Since the early 2000s, the first author had experienced more individuals presenting with increasingly complex care needs including tissue viability, incontinence, immobility and inability to self-care. Locally, a professional forum discussed relevant service developments with managers, who requested quantitative evidence to support a business case justifying change and scoping possible solutions. Subsequent searches to gather local quantitative data, or indeed published national research findings, found little evidence, especially relating to housebound individuals with high BMI and their use of community services (Williamson et al., 2020). Having identified an orphan area of both practice and research, an evaluation systematically detailing the existence of this growing population was planned. EXploring the PREvalence, Service utilisation and patient experience of Severe Obesity (EXPRESSO) planned to use mixed methods gathering quantitative data including height, weight and BMI, plus community health and social care services used. Nested qualitative work sought participants’ views on services used. As a purely observational study, no weight management intervention was included.

The local NHS organisation actively promoted research capacity-building for NMAHP staff, supporting the author to undertake the project as a part-time doctorate. This paper documents the process of gaining approvals for EXPRESSO, not outcomes of EXPRESSO itself.

## Methods

A case study approach was applied to the approvals process, to gain ‘an in-depth, multifaceted understanding of a complex issue in its real-life context’ (Crowe et al., 2011: 1). Such a case study is considered instrumental (aiding broader understanding of an issue) (Crowe et al., 2011) in that elements of this approvals process will be familiar to many researchers generally (Snooks et al., 2022). However, the real-world experience and context is infrequently documented, forgoing valuable learning, particularly for novice researchers. Thus, lessons learnt have relevance for:

(1) professionals considering service evaluation, audit or research;
(2) regulatory authorities and
(3) data analytic workstreams.

Key questions of how, what and why were used to structure exploration of the case study (Crowe et al., 2011). ‘How’ was interpreted as the processes undertaken to gain the necessary approvals, evidenced though numbers of applications, communications and clarifications, and is reported in the Results section. ‘What’ relates to specific governance requirements, such as Ethics and Caldicott processes, outlined under Context. ‘Why’ highlights the wider regulatory context, detailed in Context and Discussion sections.

### Context

Defining the context of a case study is critical to understanding it, alongside its relevance or not, to other cases (Brogan et al., 2019). Thus, pertinent contextual information is provided here. The timing of this case study was significant, spanning a period of substantial change for care sector governance, including adoption of the European Union General Data Protection Regulation (GDPR) in May 2018 (Table 1). GDPR governs processing of all personal data, giving individuals control of their data (Information Commissioner’s Office, 2021).

**Table 1.** Summary of Caldicott principles and the General Data Protection Regulation (GDPR).

Caldicott Principles^a^	GDPR
Justify the purpose	Purpose limitation
Only use personal information if it is absolutely necessary	Data minimisation
Use the minimum data required	Data minimisation
Access on a strict need-to-know basis	Data minimisation
Everyone with access to personal data should be aware of their responsibilities	Lawfulness, fairness and transparency
Comply with the law	Lawfulness, fairness and transparency
Duty to share information where needed	Integrity and confidentiality
	Accuracy

a A Caldicott Guardian is a senior individual responsible for data confidentiality within NHS and local authorities, by applying the Caldicott principles to ensure data is used properly. This covers data access, collection, storage, transfer and disposal.

Additionally, as elsewhere, Scotland has adopted a policy of health and social care integration through legally establishing Health and Social Care Partnerships (HSCPs) from April 2016. Comprising members and devolved budgets from constituent NHS and local authorities, HSCPs take responsibility for adult social care services, adult primary care, community health services and designated hospital services (Burgess, 2016).

Early EXPRESSO design involved searching Electronic Health Records (EHRs) of three to five General Practices (GPs) for individuals with recorded BMI ⩾40 kg/m^2^. Data known to be recorded in GP and wider NHS EHRs (Table 2) included number and type of services used and length of care episode, enabling some basic health economic costing. Caldicott approval is based on principles summarised in Table 2 and is essential for projects using NHS and social care data.

**Table 2.** Health and social care datasets in Scotland.^a^

Datasets	Purpose of access for clinical role	Specific data of interest for study	Data controller	Legal requirement for data gathering/sharing^b^	Legal responsibility for sign off
GP data (e.g. VISION/EMIS, Micro-test)	1. General Practice care record;	1. Height, weight, BMI	Individual GP	DSA signed off by Caldicott Guardian and GP	1. NHS Caldicott Guardian
	2. Prescribing;	2. Medications			2. GP practice
	3. Assessing home visit risk;	3. Coded comorbidities			
	4. Holistic person assessment^c^				
Trakcare (most NHS services)	1. Recording inpatient and outpatient hospital data	1. Height, weight, BMI	Health Board via Caldicott Guardian	Permission from Caldicott Guardian	3. NHS Caldicott Guardian
	2. Can link to other systems, e.g. ELMS^d^	2. Outpatient/community health episodes of care			
Social Care (SWIFT, MOSAIC, Care First)	1. PoC provision	1. PoC provision	Local Authority or since integration, delegated authority via HSCP	DSA signed off by Caldicott Guardian and Local Authority or HSCP manager	4. NHS Caldicott Guardian
	2. Occupational Therapy input	2. Occupational Therapy input			5. HSCP senior manager
	3. Social Work input	3. Social Work input			

BMI: body mass index; DSA: Data Sharing Agreement; HSCP: Health and Social Care Partnership; GP: General Practice; PoC: package of care.

a Broad principles, specifics may vary by region.

b Identifiable data for purposes other than enabling care.

c Including allergies, next of kin, key safe number.

d Equipment Loan Management Service.

Initial Caldicott approval stipulated use of a local NHS Safe Haven for data processing. Unfortunately, the associated unfunded cost (£2,000+) made this unviable. Safe Haven usage would have potentially simplified subsequent data processing by preventing the need for data transfer beyond the NHS. However, data access issues would have remained, as relevant datasets were not already within the Safe Haven.

Though the NHS lacks funding for service evaluation or research, it benefits from service providers cooperating on strategic priorities, such as excess weight. Thus, study design evolved with key collaborations:

(1) Local Intelligence Support Team (LIST) providing data analytics (Public Health Scotland, 2020);
(2) A GP practice cluster providing a population and
(3) NHS Information Technology staff for data extraction.

**Figure 1.**
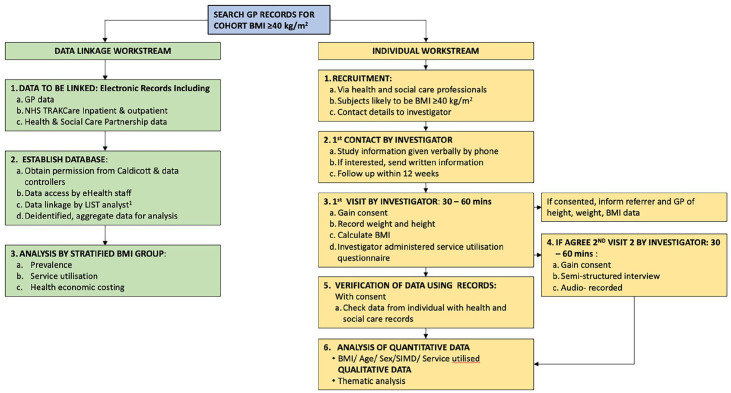
Study design February 2018: Workstream 1 (green pathway): population-level data; Workstream 2 (yellow pathway): individual-level data. ^1^Local Intelligence Support Team.

LIST staff, the Caldicott Guardian and the author met to discuss governance requirements, precipitating a second Caldicott application with amended study design (Figure 1) of two different workstreams: one exploring population-level data linkage, the other documenting individual-level data. As a doctoral study, limited data transfer to the University for supervision of analysis was essential. Caldicott approval required strict conditions on all aspects of processing to protect participants’ sensitive data.

Subsequent consideration by local NHS Research & Development staff and the Scientific Officer for the Regional Ethics Committee (NHS REC) deemed the project service evaluation rather than research, recommending organisational approval via the NHS Quality Improvement route.

### Multiple data controllers

Individual organisations control the data they record. Whilst not essential, Data Sharing Agreements (DSAs) are good practice for data sharing between organisations (Information Commissioner’s Office, 2021). They aid compliance with GDPR principles (Table 2), stipulating use of a Data Protection Impact Assessment (DPIA) where necessary. DPIAs give a framework for assessing risk regarding data processing. As with a DSA, they require review and sign off by Information Governance staff and senior managers, in addition to investigators. A thorough DPIA was undertaken, with supportive scrutiny from Data Protection staff, particularly regarding Data Protection Information for participants.

Whilst health and social care services are termed ‘integrated’, current common practice is a ‘patchwork quilt’ (Deeny and Steventon, 2015: 506) of individual data systems between NHS, local authority, third sector and independent partners. Historically these systems are problematic to integrate without major redesign and investment, thus are retained separately. Consequently, individual negotiation is required with each independent data controller regarding data sharing.

## Results

**Figure 2.**
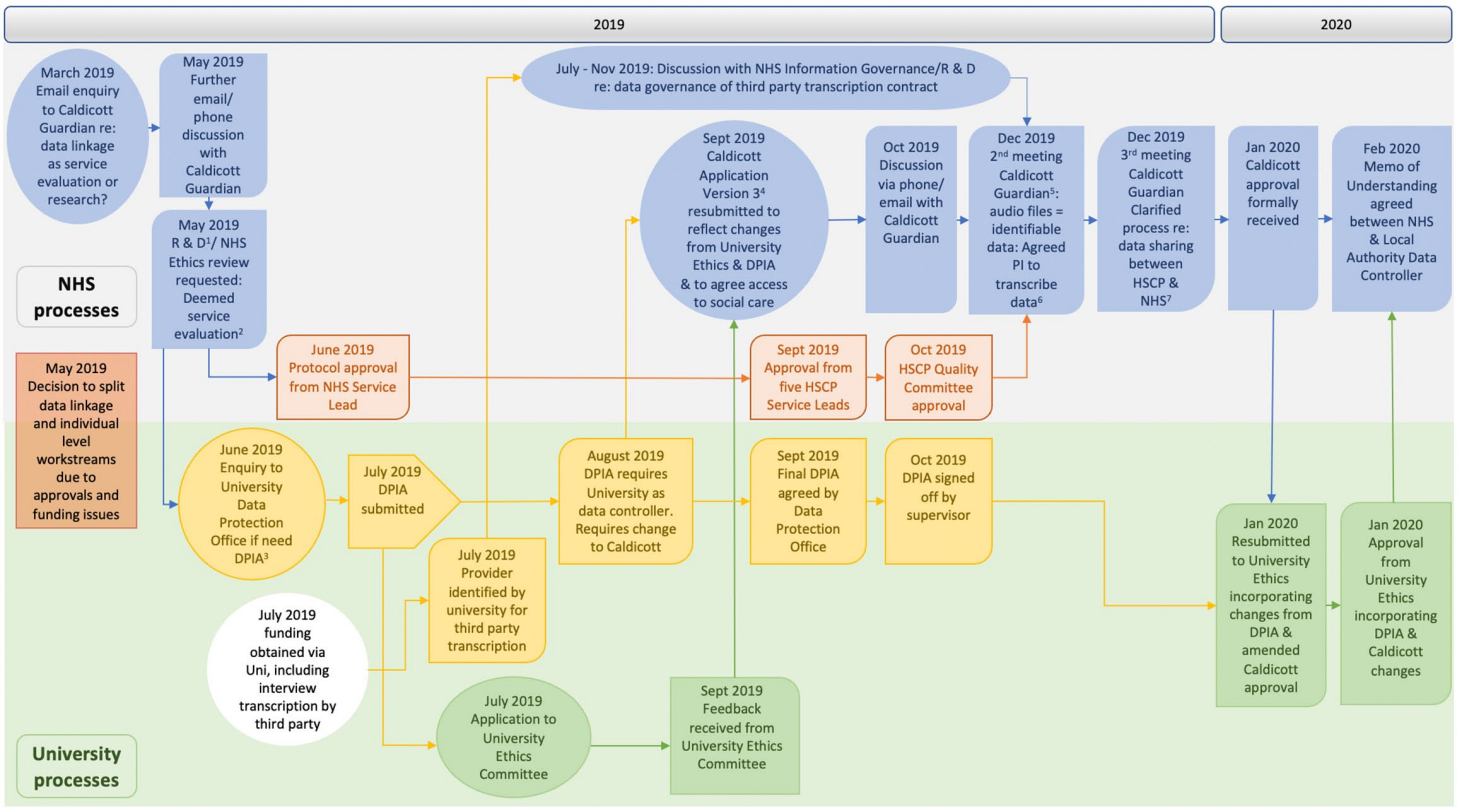
Timeline of key actions and dates of approvals March 2019 to February 2020. ^1^Research and Development Office, ^2^meaning not requiring NHS Ethics approval instead to follow University Ethics approval route, ^3^Data Protection Impact Assessment, ^4^previous two versions pre-March 2019 so not included on timeline, ^5^previous meetings pre-March 2019 so not included on timeline, ^6^three-way contracting of third party transcription between NHS, University and third party involved potentially complex data processing threatening further delay, ^7^service evaluation required assessment of overall population size but lack of shared identifiers for health and social care data gave potential for inadvertent double counting: agreed to collection of minimum dataset from both health and social care staff to exclude this.

Obtaining all necessary approvals took nearly 3 years part-time work, with two distinct ‘active’ phases: June 2017 to May 2018 and March 2019 to February 2020. Figure 2 provides a visual timeline of key actions and dates March 2019 to February 2020.

In workload terms this represented at least:

(1) 15 applications/reapplications/requests/agreements for review and sign off from nine separate stakeholders (Table 3);
(2) 20 face-to-face meetings and
(3) 225 sent emails.

**Table 3.** Stakeholders involved in required project approvals.

Approver	Organisation	Status	Procedure
Caldicott Guardian	NHS	Approver/data controller	Application
Research Ethics Committee	NHS^a^	Approver	Protocol review by officer, prior to application if needed
NHS Research & Development	NHS	Approver/sponsor	Protocol review by officer, prior to application if needed
NHS Quality Improvement	NHS	Approver	Protocol review and application
NHS Service Lead	NHS Care Provider	Data controller/sponsor/risk assessor	Protocol review
University: Ethics Committee	University	Approver/sponsor	Application & Protocol review
University: Data Protection Office	University	Data controller/risk assessor/advisor	Study data protection documents and Data Protection Impact Assessment
Health and Social Care Partnership	Joint Local Authority/NHS Care Provider	Data controller	Data Sharing Agreement/Memorandum of Understanding
General Practices	Contracted NHS Care Provider	Data controller	Data Controller Approval letter/Data Sharing Agreement

a Via the NHS Health Research Authority.

Blue colour designates NHS stakeholders; beige colour designates University stakeholders; purple colour designates other stakeholders.

Note. Please refer to the online version of the article to view the table in colour.

Although Table 3 delineates stakeholders by organisation or role, even when stakeholders were within the same organisation, they largely acted independently of each other. The most challenging areas to agree related to data sharing and transfer between organisations.

Extensive clarification was required about how to overcome lack of common identifiers between care providers and ensuring de-identification of data, both written and audio. Ongoing funding applications secured monies for transcription of qualitative, audio-recorded interview data. However, difficulty in identifying a provider with adequate data governance to satisfy the different data controllers threatened unacceptable time delay, prompting the lead author to agree to personally undertake transcription to enable the project to proceed.

Ultimately, complexity surrounding approvals contributed to separation of the two workstreams in Figure 1, allowing approvals to progress independently of each other, assuring progression of the doctoral study. The individual data workstream used consented data, simplifying data processing arrangements by removing the need for full DSAs between data controllers. Downstream data governance requirements included setting up of secure shared drives for data storage, with registration of information assets, all in addition to the above workload summary.

Factors contributing to the protracted approvals process were:

(1) Delayed responses from key approvers, whose high workload extended timescales;
(2) Negotiation with multiple data controllers creating a cyclical process, as changes required by one meant revisiting approvals already obtained and
(3) Normal evolution of a research project with study design developing in response to:
  (a) comments from approvers;
  (b) collaboration opportunities and
  (c) funding obtained.

## Discussion

The journey from being a frontline professional daily processing individual patient data, to gaining access to health and social care datasets for evaluation purposes, was longer and more complex than originally anticipated. Obesity research often concerns a single associated comorbidity, such as diabetes, or arthritis. Whilst this helps understanding of specific disease processes, it largely ignores the broader, lived experience of people with excess weight and the delivery of care by community staff. Despite holistic, person-centred care being extolled in health and social care (The Health Foundation, 2016), this project illustrates the challenges of gathering cross-sector data about the ‘whole person’ rather than a single-service or disease focus.

Complex healthcare interventions often need similarly complex evaluations, combining quantitative data analytics framed by local qualitative intelligence (Witham et al., 2015). This case study emphasises how current organisation of health and social care data systems inhibits whole system data collection on individuals. A key example is that different identifiers may be used in health and social care systems, impeding linkage (Scobie and Castle-Clarke, 2019). Such issues are solvable, for example, by seeding health or social care records with a common identifier (Witham et al., 2015), but it places further burden on staff, creating barriers to effective linkage (Heslop et al., 2020).

Encouragingly, stakeholders relatively quickly agreed in principle to share data. Delay occurred when specific technical guidance was sought from approvers. This was partly from recently introduced GDPR requirements causing a high workload for approvers, producing significant bottlenecks. Additionally, the changing data governance context meant that practical tools, such as draft templates for DSAs or Memoranda of Understanding, were largely unavailable. Since this research began, the Scottish Information Sharing Toolkit (Scottish Government, 2019) and a national Data Sharing Code of Practice (Information Commissioner’s Office, 2021) have been published, but initial progress on supportive tools was slow.

### Strengths and limitations

This project incorporated multiple strands: cross-service evaluation as a basis for person-centred quality improvement, alongside research capacity building for NMAHPs through doctoral training. On paper this was a strength, with multiple desirable elements that gave a more comprehensive understanding of the problem. Conversely in practice it was a limitation, as conflating these elements appeared confusing, even conflicting, for approvers. The United Kingdom’s Health Research Authority (HRA) decision tool (Health Research Authority, 2020b) aims to definitively classify research, yet in practice the separating line between service evaluation or research can be very fine (Committee on Publication Ethics (COPE), 2017), with approvers appearing to interpret the guidance differently. Decisions regarding project classification were often fragmented, with approvers focusing on particular aspects of the project. Collection of service level data, including client experience and lack of an intervention, led some to deem it service evaluation. Many service evaluations focus on the quality of an individual service (Twycross and Shorten, 2014). Yet lack of previous evidence, together with the observation from clinical practice, meant the aim here was on defining the current level of service provision across sectors (Twycross and Shorten, 2014). Whilst outcomes will undoubtedly inform local decision-making, the paucity of evidence in this area and the robust methodology applied, particularly to costings, mean that they are potentially indicative at a wider level. This provided substance for a doctoral research study, with findings informing the need for further research in this area, and feasibility of reaching this population. Subsequently the duality of being both a service evaluation and a PhD study has made presentation of the project to other parties more difficult. Specifically this concerned requiring transparency that approvals are for service evaluation, whilst recognising that outcomes may be of interest beyond the local area, thus warrant publication.

Undoubtedly some of the learning from this case study was from experiences common to any novice researcher. Although different projects may face diverse governance challenges, wider evidence indicates that the complexity of the health and social care data environment is challenging for many individuals and organisations involved (Higgins and Matthews, 2020; National Data Guardian, 2020). Users of the Public Benefit and Privacy Panel for Health and Social Care, which offers a governance framework for national data, report similar uncertainty and delay in accessing datasets (Lemmon, 2020).

A consultation by the National Data Guardian found staff often feel daunted in achieving compliance (National Data Guardian, 2019) with ‘bewildering’ requirements (Scottish Government, 2019: 3) making staff fearful of blame for inappropriate sharing and feeling under confident in data governance (National Data Guardian, 2019). Most recently, Health Services Research UK found governance challenges actually deter valuable research, recommending streamlined processes for low risk/non interventional studies (Snooks et al., 2022)

### Wider application

Despite huge amounts of data within the UK NHS and social care systems, data-driven innovation is not a panacea without difficulties. The COVID-19 pandemic has highlighted the underdeveloped state of social care analytics, with rudimentary data from care homes and domiciliary social care and both analytic capacity and capability disadvantaged (Bardsley et al., 2019). Data quality, particularly missing data, is a concern (Bradley et al., 2018; Higgins and Matthews, 2020). Steventon’s (2020) observation that ‘some groups suffer because their experiences are not made visible in the data’, highlights the plight of subpopulations, such as users of social care or people with severe obesity, rendered invisible through lack of reported data. Missing data tell a story precisely due to its missingness.

Practitioners can potentially offer insights from their ‘tacit knowledge’ of data collection and recording into reasons for omission (Witham et al., 2015: 236). For people with severe obesity, it can be as basic as lacking suitable scales for weighing people above 130 kg or who cannot stand. Such learning is vital if data quality and subsequent analysis is to be optimised.

Failure to establish public confidence in appropriate security and relevant usages (aside from immediate care provision) has contributed to the collapse of national health data linkage projects (Heslop et al., 2020). Thus having robust research ethics and data governance processes matters to enable public confidence. Although complexity makes public understanding of data usage a challenge, this has been acknowledged as a priority by the National Data Guardian (2019). Platform-level approaches, such as Safe Havens (Witham et al., 2015; Higgins and Matthews, 2020) and DataLoch (Usher Institute, 2020) as opposed to a project-level approach, offer a potential solution to data storage and security concerns and are being developed more widely (Heslop et al., 2020). However, evolution of these platforms takes time as they also navigate governance and dataset access hurdles. Furthermore, they can require significant funding (Higgins and Matthews, 2020) to use which may be difficult for smaller exploratory projects such as presented in this case study.

Wider solutions, such as Learning Health Systems (Scobie and Castle-Clarke, 2019) and the Health Foundation’s Improvement Analytics Unit (Bardsley et al., 2019), advocate building strong collaborations. These aim to have practitioners, public representatives, analysts and data governance staff all contributing essential components for using routine data to improve care. Regrettably many practitioners are overburdened by service demand and are infrequently available for collaboration (Sheard and Peacock, 2019) or simply lack mutual spaces to engage.

This case study observes some interesting paradoxes worth noting by practitioners seeking to evaluate routinely-collected data. Firstly, being a registered professional bound by confidentiality has minimal relevance when seeking approvals. This is because of the key difference in use of the data. If one is a direct care-provider to an individual or population, with necessary access to identifiable data, approvals for use in evaluation may potentially be more straightforward. However, here practitioners face a tension. From an evaluation perspective, direct involvement in care could lead to evaluation bias. Using a different cohort to position oneself outside the direct care team may reduce the risk of bias, but likely makes gaining of approvals more complex. Secondly, high-level approvers may possess expert data governance knowledge, but less applied knowledge of specific data systems. Consequently, practitioners can find themselves guiding approvers through the anomalies of different data systems (such as lack of common identifiers) to ensure that approvals, as given, are workable. Otherwise, it can mean revisiting approvals later, involving further delay.

The principles addressed here, in navigating a piece-meal approvals process for data governance within local government, healthcare and social care systems, are met in similar form internationally. No equivalent case report appears to have been published to critique the processes. However, comparable issues are echoed in recent reports from Europe (Haneef et al., 2020) and Australia (Palamuthusingam et al., 2019) that cite complex data governance as a barrier to data linkage for public health surveillance and research.

### Impact of COVID-19 pandemic

Data analytics have been central to the COVID-19 pandemic response, enabling near real-time data on the virus and its impact on populations. Given the global health emergency, approvers have fast-tracked applications for COVID-19 research, with the Secretary of State for Health and Social Care even simplifying processing of data without consent for a limited time (in England and Wales) (Health Research Authority, 2020a). Thus, many challenges have been circumnavigated by stakeholders meticulously working together to facilitate rapid approvals. Despite this, complexities around consent and data sharing remain, with the UK Government breaking the law by not undertaking a DPIA for the Test and Trace system (Marsh and Hern, 2020) and prestigious medical journals being forced to retract articles due to data-sharing concerns (The Lancet Digital Health, 2020). Such high-profile errors by well-resourced, expert organisations underline the convoluted intricacy of the data governance context that now exists. They exemplify why simplified guidance, such as that issued by NHSX, is helpful (Newbury, 2021).

### Recommendations

Specific issues for consideration to improve clinical research and service evaluation include:

(1) Develop further training due to the technical complexity of current data governance context.
(2) Promote toolkits such as the Scottish Information Sharing Toolkit and Data Sharing Code of Practice, including standard templates for DSAs.
(3) Encourage early, exploratory conversations with approvers regarding data governance aspects of study design.
(4) Improve resourcing of approvers: recognising the increased workload as data governance has gained complexity, so reducing waiting times for approvals.
(5) Establish clear lines of information sharing between data controllers, particularly where sharing for service benefit.
(6) Develop unified submission approach to clinically-led research and service evaluations across NHS and partner organisations.
(7) Promote clinical academic status in healthcare workforces, to release the full potential of routinely-collected data.
(8) Facilitate collaborations with practitioners on data projects.

## Conclusions

For practitioners choosing to conduct evaluation and/or research, negotiating access to the data they routinely process, can be an arduous process. Despite health and social care service integration in name, governance for health and social care data is complex and fragmented. Technically complicated data governance presents a significant barrier to enabling stakeholders to fully utilise linked data, contributing towards a risk-averse climate within relevant organisations. Yet changes wrought by the pandemic may help with striking a better balance between fully utilising data to improve care and respecting individuals’ rights.

**Key points for policy, practice and/or research**

- Practitioners’ knowledge of routinely-collected health and social care data, including what is missing, provides a potential source of great insight for evaluation and research.
- Health and social care professionals face a complex and lengthy approvals process to use data they routinely process for research or evaluation purposes.
- Current organisation of health and social care data systems inhibits cross-sector data collection and sharing on individuals, limiting evaluation of ‘whole-person’ care.
- Strong collaborations involving practitioners ‘framing’ the data could enhance evaluations.
- Practical action such as increased resources for approvers, promotion of standardised tools, streamlined processes and clear guidance for information are needed.

## Footnotes

1. The terms ‘care professional’ or ‘practitioner’ are intended to represent both health and social care professionals.
